# The Cooking and Pneumonia Study (CAPS) in Malawi: A Cross-Sectional Assessment of Carbon Monoxide Exposure and Carboxyhemoglobin Levels in Children under 5 Years Old

**DOI:** 10.3390/ijerph15091936

**Published:** 2018-09-05

**Authors:** Deborah Havens, Duolao Wang, Jonathan Grigg, Stephen B. Gordon, John Balmes, Kevin Mortimer

**Affiliations:** 1 Liverpool School of Tropical Medicine, Liverpool L3 5QA, UK; havensde@hotmail.com (D.H.); duolao.wang@lstmed.ac.uk (D.W.); 2 Centre for Genomics and Child Health, Queen Mary University of London, London E1 2AT, UK; j.grigg@qmul.ac.uk; 3 Malawi Liverpool Wellcome Trust, Blantyre, Malawi; stephen.gordon@lstmed.ac.uk; 4 Division of Environmental Health Sciences, School of Public Health, University of California, Berkeley, Berkeley, CA 94720-7360, USA; john.balmes@ucsf.edu; 5 Department of Medicine, University of California, San Francisco, San Francisco, CA 94143-0844, USA

**Keywords:** child health, environmental monitoring, personal exposure, CAPS

## Abstract

Household air pollution is estimated to cause half a million deaths from pneumonia in children worldwide. The Cooking and Pneumonia Study (CAPS) was conducted to determine whether the use of cleaner-burning biomass-fueled cookstoves would reduce household air pollution and thereby the incidence of pneumonia in young children in rural Malawi. Here we report a cross-sectional assessment of carbon monoxide (CO) exposure and carboxyhemoglobin (COHgB) levels at recruitment to CAPS. Mean (SD; range) 48-h CO exposure of 1928 participating children was 0.90 (2.3; 0–49) ppm and mean (SD; range) COHgB level was 5.8% (3.3; 0–20.3). Higher mean CO and COHgB levels were associated with location (Chikhwawa versus Chilumba) (OR 3.55 (1.73–7.26)); (OR 2.77 (1.08–7.08)). Correlation between mean CO and COHgB was poor (Spearman’s ρ = 0.09, *p* < 0.001). The finding of high COHgB levels in young children in rural Malawi that are at levels at which adverse neurodevelopmental and cognitive effects occur is of concern. Effective approaches for reducing exposure to CO and other constituents of air pollution in rural sub-Saharan African settings are urgently needed.

## 1. Introduction

Seven-hundred-million people in Africa use biomass fuels to provide energy for cooking, heating and lighting [1]. Women and young children experience high levels of smoke exposure when meals are cooked over open fires in the home due to incomplete combustion of fuel and poor ventilation [2]. The World Health Organization (WHO) estimates 4.3 million premature deaths are caused by household air pollution worldwide annually [3]. Approximately 0.5 million of these deaths are young children with pneumonia [4].

In Malawi, there is almost universal use of biomass fuels for cooking [5,6], air quality in homes is poor and personal exposure to particulate matter and carbon monoxide (CO) is high [7]. There is also a high burden of pneumonia in young children with pneumonia representing one of the most common causes of death in children under 5 years of age [8]. High levels of air pollution are thought to contribute to the high burden of childhood pneumonia in this setting.

Cleaner burning biomass-fueled cookstoves are now available that have improved combustion efficiency compared to traditional open fires. Some of the more advanced technologies (e.g., Philips fan-assisted stove) have emissions that are up to 90% and 75% lower in laboratory and field tests, respectively, than open fires [9,10]. There is evidence that particulate matter in the breathing zone around these stoves is also reduced to a similar extent [11].

Although particulate matter (PM) 2.5 is considered the “gold standard” for household air pollution monitoring, it has historically been difficult to accomplish personal PM 2.5 measurements, particularly in children. CO is easier to measure and has been used as a marker for exposure to products of combustion as it is a major component of household air pollution [12], and has been shown to correlate well with PM 2.5 in specific situations such as: Use of single biomass cookstoves, exclusive biomass users, and in rural settings, as found in Malawi [13]. Carboxyhemoglobin (COHgB) is a biomarker for CO and is formed as CO binds with the hemoglobin molecule. It represents both the environmental exposure to CO coupled with the specific physiologic effects within the individual [14].

In the setting of a cookstove intervention trial in rural Malawi, we obtained baseline (i.e., prior to the intervention) personal CO exposure and COHgB levels in a large sample of children participating in the trial. We report these data to provide evidence of the background exposure to household air pollution of children living in rural sub-Saharan Africa.

## 2. Materials and Methods

### 2.1. Setting

We conducted the Cooking And Pneumonia Study (CAPS), a community-level cluster randomized controlled trial (*n* = 150 communities; *n* = 8626 households; *n* = 10,750 children) to determine whether a cleaner burning biomass-fueled cookstove intervention would reduce pneumonia incidence in children under 5 years of age living in rural Malawi [15]. Intervention households received two cleaner burning cookstoves (Philips HD4012LS; Philips South Africa, Johannesburg, South Africa) and a solar panel for charging when they were enrolled, with education and cookstove/solar panel repairs/replacements as needed. Control households received the same items at the end of the 2-year study [15].

This cross-sectional study was conducted within the context of the larger CAPS trial in two rural districts in Malawi: Chikhwawa, an impoverished area 60 km south of the nearest urban center (Blantyre, Malawi) near the Shire River in the South, that experienced droughts, flooding and famine during the time of the study; and Chilumba, an agrarian and fishing community on the shore of Lake Malawi that is 75 km from the nearest urban center (Karonga, Malawi) in the North. Recruitment of participating households in both Chikhwawa and Chilumba primarily occurred during the dry season, but ongoing enrollment also occurred if new participants came within the catchment area during the trial period. Homes in these rural areas tended to be made of brick and to consist of one main room divided by partitions. Cooking occurred within the home, which may have had limited ventilation, or be outdoors, often under a veranda or in an area that may have partial walls or roof. The rainy season in this region generally encompasses November through February with a slightly cooler season from June through August. The remainder of the year is routinely hot and dry.

### 2.2. Participants

All children with parental or guardian consent aged up to 4.5 years living in the clusters across the two districts were eligible to participate in CAPS. Recruitment took place between 9 December 2013 and 28 February 2016. All participants in CAPS, including those that became eligible during the course of the trial, through movement into the catchment area or as new births, were also potentially eligible for this sub-study. The primary CAPS outcome was WHO Integrated Management of Childhood Illness (IMCI)-defined pneumonia in children under the age of 5 and children were enrolled up to the age of 4.5 in order to obtain at least 6 months of data on each participant. At the time of enrollment, a random number was created and children who fell within a predetermined range were recruited with the aim of randomly selecting 1 in 4 CAPS participants for this sub-study. The larger CAPS sample size was designed to have 90% power to detect a 20% difference in the pneumonia incidence rate between the intervention and control groups assuming a baseline rate of 5 per 100 child years. The 25% sample size for the CO monitoring sub-study was based on feasibility, given the large number of children recruited for CAPS.

### 2.3. Data Collection (Questionnaires)

Fieldworkers trained using study-specific standardized operating procedures attended the homes of all participating children to interview their parents or guardians at the baseline data collection visit for CAPS. Electronic Case Report Forms (CRFs) programmed using Open Data Kit (ODK) software and administered offline using smart phones (Samsung Galaxy S3, Samsung, Seoul, Korea) were used to collect data. Demographic, socioeconomic, health and household air pollution data were collected in this way at enrolment. An additional questionnaire was administered at the time of collection of CO monitors (see below) about potential smoke exposures and cooking practices including number of meals, fuel used and cooking location during the time CO monitors were worn.

### 2.4. Data Collection (Carbon Monoxide Exposure Assessments)

**Figure 1:**
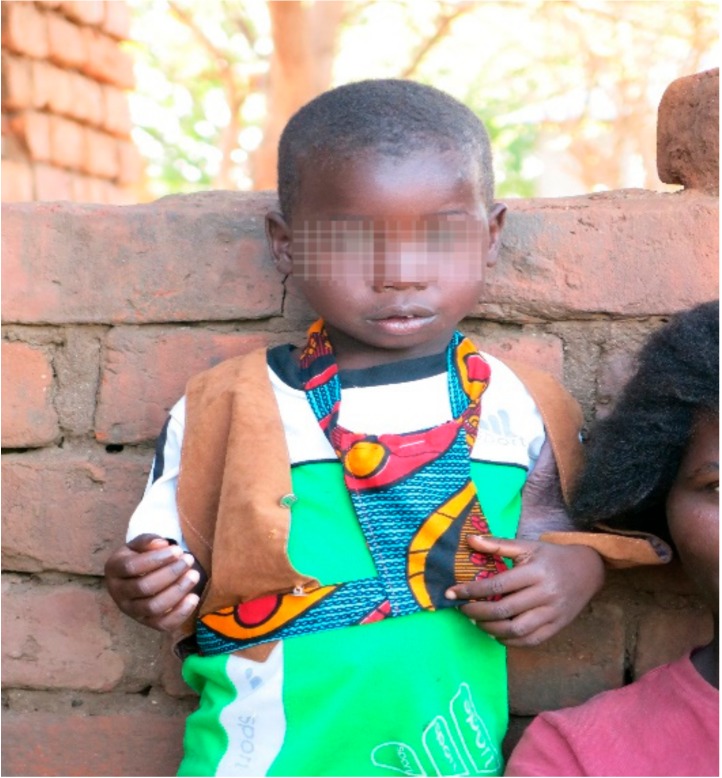
Picture of child wearing Lascar CO monitor.

Portable CO monitors (EasyLog-USB-CO Lascar Electronic Ltd., Salisbury, UK) were used to conduct continuous, real-time monitoring of exposure to CO. Measurements were taken at 30-s intervals. Lascar monitors have a measurement range of 0–1000 ppm with an accuracy of ±6% of the reading. They can operate within a temperature range of −10 to +40 °C. Field workers utilized laptop computers to initiate the monitors at the time of arrival at the participants’ homes. The participants were instructed to wear the monitors at all times during the 48-h monitoring period, with the exception of when the child was sleeping, there was rain, or if the monitor could be exposed to water through swimming or bathing. Monitors were removed at night and put beside the child. The Lascar monitors were placed in a cloth bag made of local fabrics that was attached around the child’s neck with Velcro (Figure 1). CO data files were downloaded from the Lascar monitors at the time of pick up from the participants.

At the time of monitor collection, transcutaneous COHgB measurements were obtained on the children (Rad-57 Rainbow SET Pulse CO-Oximeter; Masimo, Irvine, CA, USA). These monitors have a range of 1–40% and can operate within a temperature of −18 to +54 °C. Prior to use each day, the COHgB monitor was checked with the manufacturer’s testing device (Masimo Rainbow Tester) to confirm accuracy. Three COHgB measurements were obtained on each child, ideally on three separate digits. As the precision of the monitor is ±3%, if the range of results was greater than 6% then an additional measurement was obtained. A pediatric sensor that was suitable for children aged 6 months and above was used; children younger than 6 months were not eligible for COHgB measurements and, therefore, only had CO monitoring.

### 2.5. Data Management

Data were transferred from the smart phones and laptops to a secure server at each study site and then onwards to a central secure server at the Liverpool School of Tropical Medicine for cleaning and preparation for analysis.

### 2.6. Statistical Methods

R software was used to process the CO monitor measurements. CO monitor files with greater than 5790 measurements were truncated at the first 48 h of assessment. Files containing fewer than 500 measurements were excluded. Files with incorrect data and non-plausible graphs were removed for quality control. STATA (Version 14) was utilized for the overall analysis including the questionnaire results. Multilevel logistic regression modeling, taking cluster and household effects into account, was used to identify factors associated with higher CO and COHgB. Higher CO and higher COHgB were defined as greater than the median for the sample. The regression analysis was a two-step process: Univariable analysis was performed initially and variables having associations with *p* < 0.05 were included in a multivariable analysis. T tests were used to compare group means. Log means were utilized for analyses requiring a normal distribution. Chi-squared tests were used for categorical outcomes. Spearman’s rho was used for correlation between nonparametric values. A *p* value < 0.05 was considered statistically significant.

### 2.7. Ethical Considerations

The study was approved by the College of Medicine Research Ethics Committee in Malawi (Ref P.11/12/1308) and the Liverpool School of Tropical Medicine Research Ethics Committee in the UK (Ref 12.40). Parents and guardians of children gave written informed consent prior to participation. When needed, information sheets and consent forms were read out with witnessed thumbprints taken in place of signatures. The CAPS protocol was peer reviewed and published by *The Lancet* [16]. Trial registration ISRCTN 59448623.

### 2.8. Role of the Funding Source

The funders had no role in the study design, data collection, analysis, interpretation or writing of the report. The corresponding author had full access to all the study data and had final responsibility for the decision to submit for publication.

## 3. Results

A total of 10,750 participants (4242 Chikhwawa; 6508 Chilumba) from 8550 households and 150 communities were recruited into CAPS. Mean age (SD) was 24 (16) months and 50.7% were female (Table 1).

**Figure 2:**
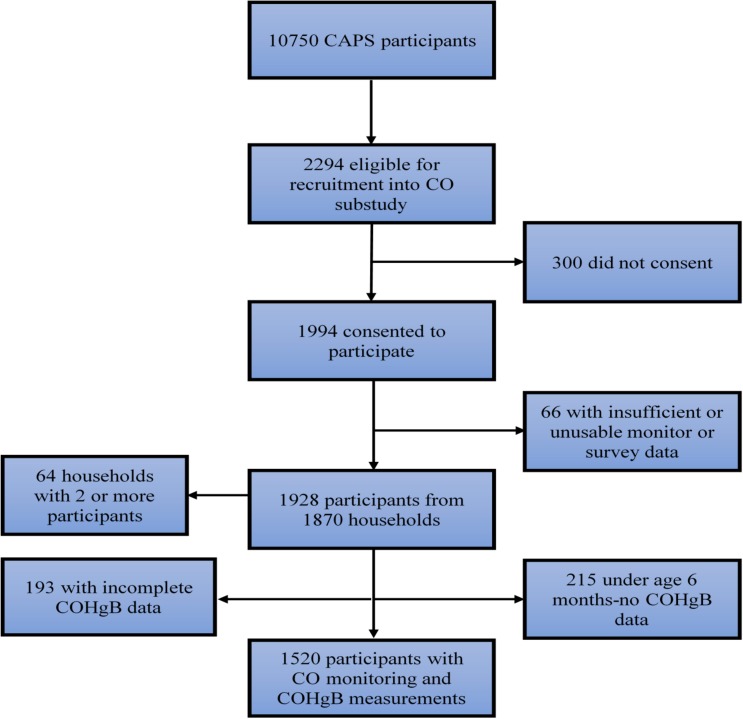
Trial profile.

We randomly selected 2294 of these participants for inclusion into this sub-study and 1994 consented to participate. Of these, 1928 children (823 Chikhwawa, 1105 Chilumba) from 1870 households contributed data for analysis; 24 participants did not enter the study in the end and 42 participants had insufficient or unusable data. COHgB measurements were obtained from 1520 children (Figure 2). The mean (SD) age of the participating children was 25 (15) months and 50.1% were female.

### 3.1. Study Sites

There were socioeconomic and lifestyle differences in participants from Chilumba and Chikhwawa with more indicators of poverty in Chikhwawa: Open defecation was used for toileting by 31% of the Chikhwawa participants while 93% of children from Chilumba had at least simple pit latrines; 6.9% of Chikhwawa participants reported a formal water access point, while 75% in Chilumba had access to a bore hole for water and 36% to a household or communal tap. A significantly larger percentage of Chikhwawa participants were in households where they lacked sufficient food (77% vs. 36%) and money for soap (83% vs. 52%) compared to Chilumba (*p* < 0.001).

### 3.2. Pneumonia and Other Reported Symptoms

At baseline, 61% of parents/guardians reported that their child had experienced a cough in the 3 months prior to enrollment, 17% had wheezing, 6% a burn and 16% a diagnosis of pneumonia in the 12 months prior to enrollment. Fewer wheezing symptoms (14%) were reported in Chikhwawa compared to Chilumba (20%) (χ^2^ = 14, *p* < 0.001).

### 3.3. Cooking Location

Few households cooked inside their main home in the dry season, with only 3% reporting cooking in a kitchen and another 3% reporting cooking inside within a living area. Many households (40%) reported cooking outside in a separate walled and roofed structure in the dry season, 14% reported cooking outside in a structure with a roof only, 20% cooked on a veranda, and 23% reported cooking in the open air. In the rainy season, less than 1% would attempt to cook outside in the open air, 12% outside under a roof, 44% outside in a structure with roof and walls, with 23% reporting cooking on a veranda, 9% inside in a kitchen and 14% inside in a living area.

### 3.4. Cooking Events

Survey data on 10,821 individual cooking events were collected. Respondents reported a mean (SD) of 5.54 (1.11) cooking events during the 48-h monitoring period. Although the majority of households did not receive the Philips stove until the monitoring had been completed, a small number did have them due to the logistics of stove delivery in this rural area and the ongoing enrollment process. If a Philips stove was available, the participating household was asked not to use it until the monitoring period was completed, although a small number (1%) of the cooking events were reported as utilizing the Philips stove. Most individual cooking events were reported as lasting 1–2 h (46%), with 39% lasting 30–60 min, 10% lasting 2–3 h, fewer than 5% lasting less than 30 min, and fewer than 1% lasting over 3 h.

### 3.5. Fuels and Stoves Used

The majority of households cooked most often with wood (58%) over an open fire (60%); 17% reported using charcoal, with 5% indicating they owned a charcoal burner. Less than 1% of households reported the use of any other type of improved cookstove at baseline.

### 3.6. Other Sources of Smoke Exposure

Many participants reported extraneous exposures to smoke not from cooking, with the majority noting another source of exposure, including burning rubbish in the village (42%), cooking for others as a business (14%), brick making (5%), and use of kerosene lamps (2%). The presence of one or more smokers was noted in 17% of the households.

### 3.7. CO Exposure Monitoring

**Figure 3:**
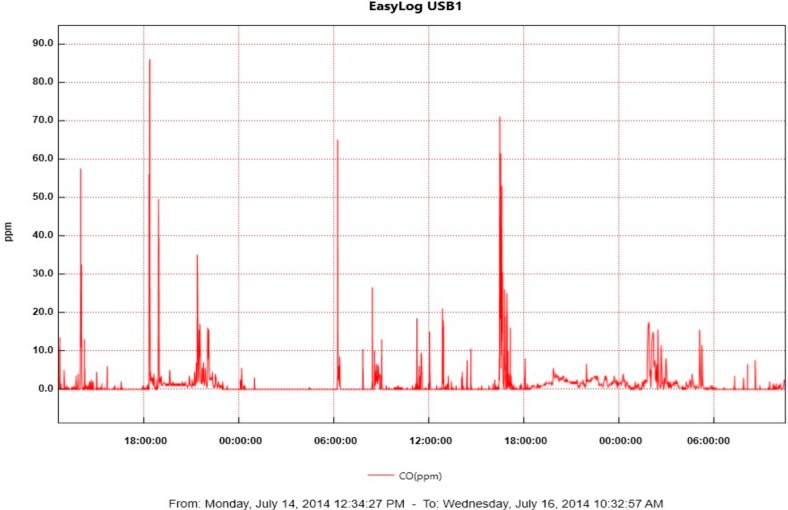
Example of CO exposure graph from Lascar CO monitor.

CO exposure monitoring data were obtained from 1928 participants (See Figure 3 for example of CO exposure graph).

Overall mean CO exposure was 0.90 ppm (SD = 2.3; range 0–49). The median CO was 0.50 ppm with an interquartile range (IQR) of 0.21–1.00. Only 20 participants had average CO levels above the WHO recommended 24-h CO exposure level of 7 ppm; however, 51 participants experienced CO exposures over the recommended 15-min exposure level of 100 ppm and 120 participants had 1-h averages greater than the recommended level of 10 ppm [17].

Self-reported burns (OR 1.63 (1.02–2.60)), living in Chikhwawa vs. Chilumba (OR 7.11 (4.11–12.28)), and cooking inside in the dry (OR 2.13 (1.36–3.36)) and rainy seasons (OR 1.38 (1.07–1.78)) were associated with CO levels higher than the median. Poverty as evidenced by lack of food (OR 1.91 (1.44–2.54)) and lack of money to buy soap (OR 1.57 (1.20–2.06)) were also associated with higher CO levels. In multivariable analysis, occurrence of burn (OR 1.58 (1.00–2.50)), lack of food (1.50 (1.14–1.95)), and living in Chikhwawa vs. Chilumba (OR 3.55 (1.73–7.26)) were independently associated with having a higher CO level after controlling for other factors (Table 2).

Mean (SD) CO exposure was higher in Chikhwawa (1.27 ppm (2.79)) versus Chilumba participants (0.62 ppm (1.79)). Higher mean (SD) CO levels were noted as households cooked in more confined areas during the dry season: outside under a roof only, 0.56 ppm (0.92); veranda, 1.39 (2.97); inside in kitchen, 1.83 ppm (3.64); and inside in a living room 1.75 ppm (2.52). Similar levels were noted for the cooking areas during the rainy season: outside under a roof only, 0.52 ppm (0.90); veranda, 1.19 (2.74); inside in kitchen, 1.65 ppm (5.71); and inside in a living room, 1.16 ppm (1.52).

To assess exposures during potential cooking events, maximum 15-min, 30-min, 60-min and 90-min exposures were determined for each participant. Mean (SD) CO levels were 23.0 ppm/minute (29.0) for the maximum 15-min intervals, 16.8 ppm/minute (23.8) for maximum 30-min exposures, 12.0 ppm/minute (19.0) for maximum 60-min exposures, and 8.6 ppm/minute (13.4) for the maximum 90-min exposures.

### 3.8. COHgB

COHgB was measured in 1520 participants; 215 were under 6 months of age and 193 had incomplete data. Mean (SD) COHgB was 5.8% (3.3) and ranged from 0 to a maximum of 20.3%. Median COHgB was 5.5% (IQR = 3.3–7.7). A 4th COHgB measurement was taken in 531 (35%) of the children. There was a statistically significant difference in the mean (SD) COHgB between children who required three vs. four measurements (5.4% (3.5) vs. 6.6% (2.9), *p* < 0.001), in Chikhwawa versus Chilumba participants, (6.9% (3.7) vs. 5.0% (2.8), *p* < 0.001) and in males versus females (6.0% (3.3) vs. 5.6 (3.3), *p* = 0.03).

Male gender (OR 1.35 (1.02–1.79)), living in Chikhwawa (OR 3.12 (1.89–5.14)) and poverty as evidenced by lack of food (OR 1.47 (1.09–1.99)) were associated with COHgB levels higher than the median. In multivariable analysis, only living in Chikhwawa independently remained a positive association (OR 2.77 (1.08–7.08)) (Table 3).

### 3.9. Relationship between CO and COHgB Measurements

Mean COHgB levels were weakly positively associated with mean CO levels (Spearman’s ρ = 0.09, *p* < 0.001), CO total exposure (Spearman’s ρ = 0.10, *p* < 0.001) and the mean CO level obtained during the final 6 h of monitoring, just prior to COHgB testing (Spearman’s ρ = 0.10, *p* < 0.001). Mean COHgB levels did not correlate with overall maximum CO exposures (Spearman’s ρ = 0.03, *p* = 0.32), maximum 15-min (Spearman’s ρ = 0.02, *p* = 0.45), maximum 30-min (Spearman’s ρ = 0.21, *p* = 0.42), maximum 60-min (Spearman’s ρ = 0.03, *p* = 0.21) or maximum 90-min exposures (Spearman’s ρ = 0.01, *p* = 0.58).

## 4. Discussion

In this sub-study of children participating in the CAPS randomized controlled trial in rural Malawi, we measured personal exposure to CO among 1928 children under the age of 5 at the time of enrollment. While exposures averaged over 48 h were not high, peaks in exposure were frequent. In contrast to the average personal CO exposure levels, COHgB levels were high (5.8%) and at levels commonly associated with tobacco smoke exposure. Personal exposure to CO correlated poorly with COHgB levels.

**Figure 4:**
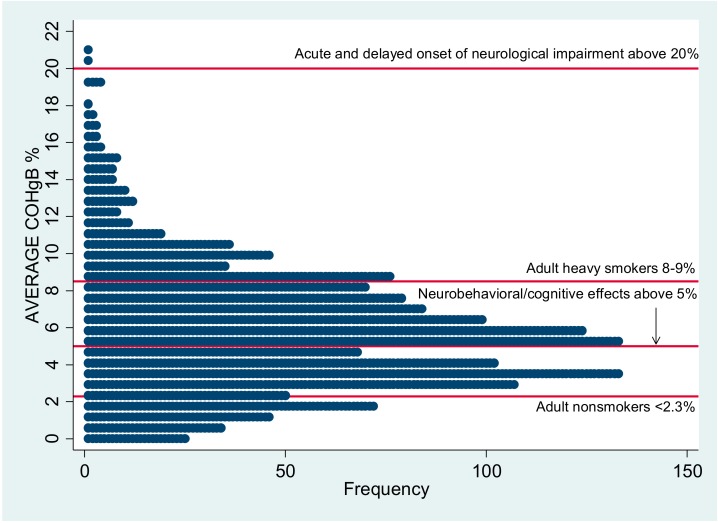
Dotplot showing frequency of participant average COHgB% in relation to ATSDR reference levels. Reference values: Agency for Toxic Substances and Disease Registry 2012.

The mean 48-h CO exposure of 0.90 ppm that we report for rural Malawian children is consistent with levels noted in other household air pollution research from developing countries: 0.93–2.19 ppm in Guatemala [18]; 1.04 ppm in Banjul, Gambia [19]; 3.3 ppm in Burkina Faso [20]; 0.82–5.57 ppm in Haryana, India [21]; 2.35 ppm in Mexico [22]; and 1.87 ppm in another study in rural Malawi [7], although methodologies vary. The mean COHgB level reported here, 5.8%, is well above the 1–2% level considered as background in urban non-smokers [23]. It is concerning that the average COHgB is above 5%, which, as illustrated in Figure 4, is the level at which neurobehavioral and cognitive effects may be seen in children and a level at which there is decreased exercise tolerance in healthy adults and an increased risk for cardiovascular arrhythmias and events in individuals with underlying coronary artery disease [24].

Other studies of COHgB associated with biomass fuel use have shown results from 15.74% in India due to cooking events [25] and 12–14% in Guatemala in association with the wood-fired *temazcal* [26]. At a steady state, utilizing the predicted values from the Coburn-Forster-Kane model (adults), a COHgB of 5.8% might correspond to CO exposure between 20–40 ppm, which suggests that fairly high peaks of CO exposure from cooking or other sources may be present in the environment [24]. However, exposures due to household air pollution could be quite variable and the subsequent rate of elimination of COHgB, although traditionally described as having a half-life of 2–6 h [14], would represent a complex process, affected by ongoing exposures, exercise, rate of respiration, age and gender [24].

There have been few studies in Africa that have compared CO and COHgB levels within the context of household air pollution and none that have used non-invasive COHgB testing. In this study, there was not a good correlation between these two measures, a finding that has been seen in other long-term environmental CO monitoring studies [27]. Lam et al. also noted difficulties in correlating solid fuel associated CO exposure with COHgB particularly at lower levels [26], and in larger scale clinical testing scenarios, accuracy, precision, and correlation with CO levels as measured by the Rad-57 have been variable with reports of both over- and under-estimation [28]. The poor association in this study is likely due to a number of reasons, including the complex kinetics of COHgB and fluctuating exposure to CO with COHgB reflecting more recent exposure, participants’ levels of exercise and activity, possible lack of precision in COHgB measurements due to field conditions or user error, possible poor compliance with wearing the Lascar monitor, and the lack of accuracy of co-oximeter testing at low levels of CO exposure [23,29]. Endogenous COHgB formation, as can be seen with inflammation and severe anemia, could also be a factor [30].

Based on the results of the questionnaires, CO levels and COHgB in this study represent not only exposures from biomass cooking, but also potentially from many other sources of combustion, although the self-reported exposures to these other sources were not significantly associated with either personal CO or COHgB in the multivariable analysis. Exposure to burning rubbish was frequently reported as was exposure to burning of agrarian waste, which may be burned for extensive periods of time to prepare fields for future crops and to remove small pests. Chikhwawa is also situated next to Malawi’s largest sugar cane grower, whose large fields were routinely burned prior to harvesting. Making beer at home is also a lengthy process requiring 6–8 h of boiling for distillation of each pot. Personal CO exposures from cooking also reflect the frequent occurrence of outdoor and open-air meal preparation, particularly in the dry season, which is the majority of the year in Malawi. Exposure assessment that could clarify the contributions of various combustion sources would assist in avoiding future interventions that may have limited value in decreasing household air pollution.

This sub-study of CAPS has several strengths. It is the largest study to date to measure personal exposure to markers of biomass smoke of young children in Africa. The use of real-time monitors for CO provided the ability to obtain detailed exposures, including the assessment of peak levels, over a 48-h monitoring period. The non-invasive measurement of COHgB added a specifically relevant biomarker of exposure. Limitations include likely information bias in terms of questionnaire ascertainment of cooking behavior. There was a low number of positive responses in the Chikhwawa population with respect to household characteristics such as type of fuel used, suggesting problems with the questionnaire or its administration by fieldworkers as many households did not report cooking with any fuels or having any toileting options. Although all field workers were trained and all surveys piloted extensively, Chikhwawa was the first site of enrollment and data acquisition improved over time. The lack of a neonatal sensor also led to the exclusion of COHgB readings on the youngest children.

## 5. Conclusions

The use of biomass fuels is ubiquitous in rural Malawi. Exposures to smoke occurs commonly from a range of sources including household cooking, commercial cooking, rubbish and agricultural field burning. The COHgB levels we observed in young children in rural Chikhwawa were high and at levels associated with adverse health outcomes including adverse neurobehavioral and cognitive effects. We conclude that children in settings like those in rural Malawi in Africa are likely exposed to potentially harmful levels of CO from multiple sources and intervention with cleaner-burning biomass-fueled cookstoves alone may not be sufficient to deliver improved health outcomes. More comprehensive environmental interventions that substantially improve air quality are likely needed to achieve this.

## Figures and Tables

**Table 1:** Baseline characteristics.

	CAPS (Overall)	CO Sub-Study
**Community Characteristics**	10,750	1928
Chilumba	6508 (60.5%)	1105 (57.3%)
Chikhwawa	4242 (39.5%)	823 (42.7%)
**Participant Characteristics**		
Age (mean)	24 mo	25 mo
Sex		
Female	5445 (50.7%)	966 (50.1%)
Male	5305 (49.3%)	962 (49.9%)
Cough in past 3 months	6259 (58.2%)	1170 (60.7%)
Wheezing in past 3 months	1770 (16.5%)	334 (17.3%)
Cooking related burn in past 3 months	516 (4.8%)	118 (6.1%)
Diagnosis of pneumonia in past 12 months	1636 (15.2%)	317 (16.4%)
**Household Characteristics**		
Number of households	8550	1870
Cooking Fuel		
Wood	4946 (57.8%)	1089 (58.2%)
Crop Residue	2686 (31.4%)	657 (35.1%)
Charcoal	1310 (15.3%)	317 (17.0%)
Dung	45 (0.5%)	11 (0.6%)
Electricity	19 (0.2%)	2 (0.1%)
LPG	2 (0.0%)	0
Kerosene	8 (0.1%)	2 (0.1%)
Other	2 (0.0%)	3 (0.2%)
Smoker in the household	1443 (16.9%)	313 (16.7%)
Experienced a time in the past year when there was not enough money for food	4347 (50.8%)	1038 (55.5%)
Experienced a time in the past year when there was not enough money to buy soap	5317 (62.2%)	1280 (68.4%)
**Daily or almost daily exposure to smoke from:**		
Burning rubbish	3471 (40.6%)	791 (42.3%)
Cooking for others as a business	1083 (12.7%)	268 (14.3%)
Making beer	100 (1.2%)	21 (1.1%)
Making bricks	395 (4.6%)	97 (5.2%)
Kerosene lamps	195 (2.3%)	47 (2.5%)
Mosquito coils	128 (1.5%)	29 (1.6%)
Other sources	111 (1.3%)	26 (1.4%)
**Primary cooking location—Dry season**		
Outside with a separate structure with a roof only	1160 (13.6%)	258 (13.8%)
Outside in a separate structure with a roof and walls	3331 (39.0%)	756 (40.4%)
Outside in the open air	1827 (21.4%)	422 (22.6%)
Outside on the veranda (khonde)	1673 (19.6%)	366 (19.6%)
Inside in a separate room (kitchen)	355 (4.2%)	63 (3.4%)
Inside in a living room	286 (3.3%)	63 (3.4%)
**Primary cooking location—Rainy season**		
Outside with a separate structure with a roof only	1076 (12.6%)	229 (12.2%)
Outside in a separate structure with a roof and walls	3613 (42.3%)	816 (43.6%)
Outside in the open air	87 (1.0%)	19 (1.0%)
Outside on the veranda	1981 (23.2%)	434 (23.2%)
Inside in a separate room (kitchen)	701 (8.2%)	160 (8.6%)
Inside in a living room	1174 (13.7%)	270 (14.4%)

**Table 2:** Association between household characteristics and elevated CO levels (*n* = 1928) ^1^.

	Univariable	Multivariable
	OR (95% CI) ^2^	OR (95% CI)
**Presence of cough in 3 months prior**	**0.78 (0.63–0.97)**	**0.77 (0.62–0.96)**
Diagnosis of pneumonia in 12 months prior	0.83 (0.63–1.10)	
Presence of wheeze in 3 months prior	1.17 (0.89–1.53)	
**Occurrence of burn in 3 months prior**	**1.63 (1.02–2.60)**	**1.58 (1.00–2.50)**
Wood	**0.17 (0.14–0.21)**	0.67 (0.36–1.24)
Charcoal	0.73 (0.53–1.02)	
Crop	**0.55 (0.42–0.73)**	0.98 (0.75–1.27)
Dung	0.25 (0.05–1.30)	
Other	2.51 (0.20–31.14)	
Smoker in the home	1.15 (0.86–1.55)	
Male gender	1.01 (0.82–1.24)	
Making bricks	0.70 (0.42–1.17)	
Making beer	1.20 (0.46–3.11)	
Rubbish burning	**0.45 (0.31–0.67)**	0.93 (0.70–1.23)
Use of kerosene lamp	0.73 (0.36–1.47)	
Use of mosquito coil	0.87 (0.38–2.02)	
Cooking as a business	0.76 (0.55–1.05)	
**Period without food in the prior year**	**1.91 (1.44–2.54)**	**1.50 (1.14–1.95)**
Period without money to buy soap in the prior year	**1.57 (1.20–2.06)**	1.02 (0.79–1.32)
**Chikhwawa vs. Chilumba**	**7.11 (4.11–12.28)**	**3.55 (1.73–7.26)**
Cooking inside in the dry season	**2.13 (1.36–3.36)**	1.56 (0.93–2.62)
Cooking inside in the rainy season	**1.38 (1.07–1.78)**	1.04 (0.78–1.38)

^1^ Elevated OR defined as greater than the median mean CO of 0.5 ppm. ^2^ Statistically significant associations in bold.

**Table 3:** Association between household and participant characteristics and elevated carboxyhemoglobin levels (*n* = 1520) ^1^.

	Univariable	Multivariable
	OR (95% CI) ^2^	OR (95% CI)
Presence of cough in 3 months prior	0.97 (0.75–1.27)	
Diagnosis of pneumonia in 12 months prior	1.03 (0.76–1.40)	
Presence of wheeze in 3 months prior	1.34 (0.95–1.90)	
Occurrence of burn in 3 months prior	1.06 (0.65–1.73)	
Wood	**0.34 (0.21–0.55)**	0.82 (0.36–1.89)
Charcoal	**0.59 (0.38–0.93)**	0.88 (0.59–1.31)
Crop	**0.58 (0.40–0.85)**	1.07 (0.74–1.55)
Dung	0.63 (0.10–3.77)	
Smoker in the home	1.27 (0.89–1.81)	
Male gender	**1.35 (1.02–1.79)**	1.30 (0.99–1.72)
Making bricks	0.71 (0.39–1.29)	
Making beer	0.61 (0.18–2.13)	
Rubbish burning	**0.56 (0.39–0.81)**	1.09 (0.75–1.59)
Use of kerosene lamp	1.53 (0.65–3.56)	
Use of a mosquito coil	0.68 (0.24–1.99)	
Cooking as a business	1.05 (0.71–1.57)	
Period without food in the prior year	**1.47 (1.09–1.99)**	1.05 (0.79–1.40)
Period without money to buy soap in the prior year	1.21 (0.90–1.62)	
**Chikhwawa vs. Chilumba**	**3.12 (1.89–5.14)**	**2.77 (1.08–7.08)**
Cooking inside in the dry season)	1.09 (0.67–1.79)	
Cooking inside in the rainy season	0.69–1.30)	

^1^ Statistically significant associations in bold. ^2^ Elevated OR defined as greater than median COHgB 5.5%.
